# Successful embolization of a ruptured pancreaticoduodenal artery aneurysm associated with the median arcuate ligament syndrome

**DOI:** 10.4103/0971-3026.40305

**Published:** 2008-05

**Authors:** Jin Iwazawa, Masao Hamuro, Yukimasa Sakai, Kenji Nakamura

**Affiliations:** Department of Radiology, Nissay Hospital, 6-3-8 Itachibori, Nishiku, Osaka, Japan; 1 Department of Radiology, Osaka City, University Graduate School of Medicine, 1-4-3 Asahimachi, Abenoku, Osaka, Japan

Celiac axis compression caused by the median arcuate ligament (MAL) syndrome is a rare entity. Occasionally, the resultant celiac axis stenosis increases blood flow in the pancreaticoduodenal arcade, and the consequent volume overload can lead to arterial dilatation and aneurysm formation.[1] Recently, transcatheter embolization has become an alternative initial treatment for ruptured aneurysms.[2] Previously reported cases have indicated that ruptured aneurysms should be isolated by coils or other embolic agents.[3] However, efforts at such isolation carry the potential risk of re-bleeding if the ruptured aneurysm has other arterial communications.

## Case Report

**Figure 1:**
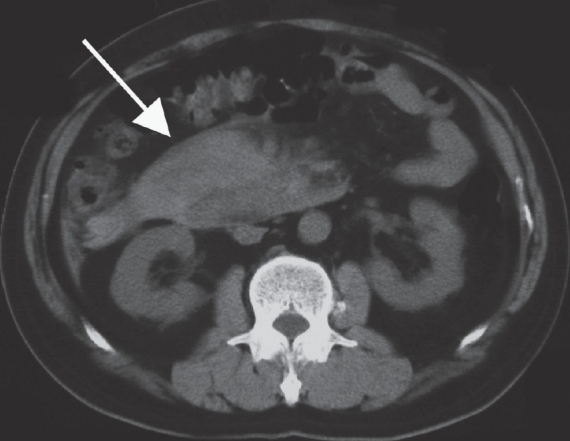
Plain axial CT scan shows a massive retroperitoneal hematoma (arrow) around the pancreatic head

**Figure 2:**
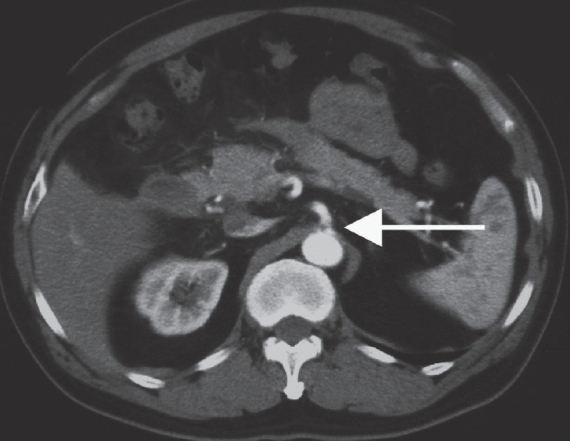
Contrast-enhanced CT scan shows a prominent median arcuate ligament (arrow) compressing the origin of the celiac axis

**Figure 3:**
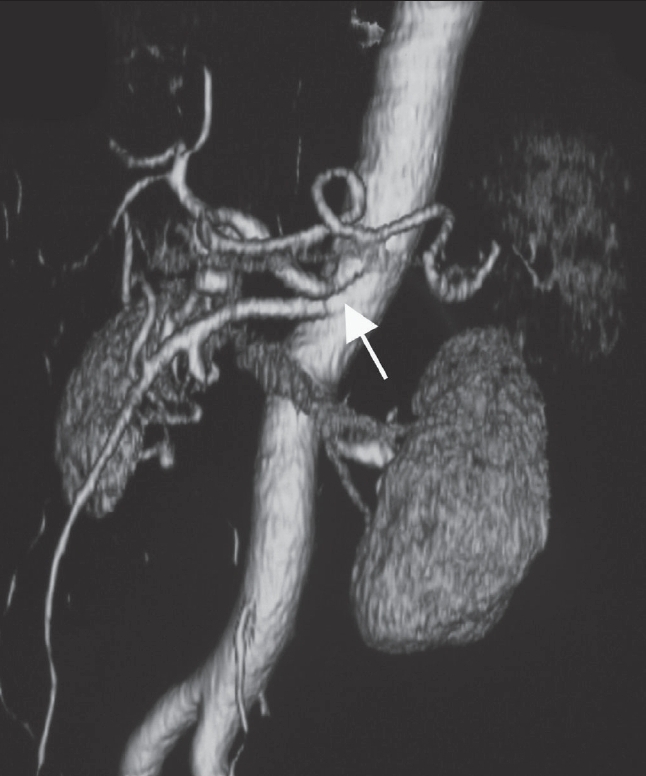
Anterolateral view of a three-dimensional CT aortogram shows stenosis of the celiac axis (arrow)

A 62-year-old man with no prior history of gastrointestinal disease was referred to our hospital with acute abdominal pain. The pain had started 2 days prior to admission and had gradually increased. He denied alcohol use or prior surgery. On examination, his blood pressure and pulse rate were 90/60 mmHg and 104/min, respectively. Blood tests showed an elevated white cell count (9900/mm^3^) and C-reactive protein level (5.77 mg/dl) as well as decreased serum hemoglobin (9.6 g/dl). CT scan revealed a massive retroperitoneal hematoma around the pancreatic head [Figure 1]. Contrast-enhanced images showed extrinsic compression of the celiac axis by a MAL [Figure 2] that caused severe stenosis of the celiac axis [Figure 3]. No obvious aneurysm or diseased arteries were found.

**Figure 4:**
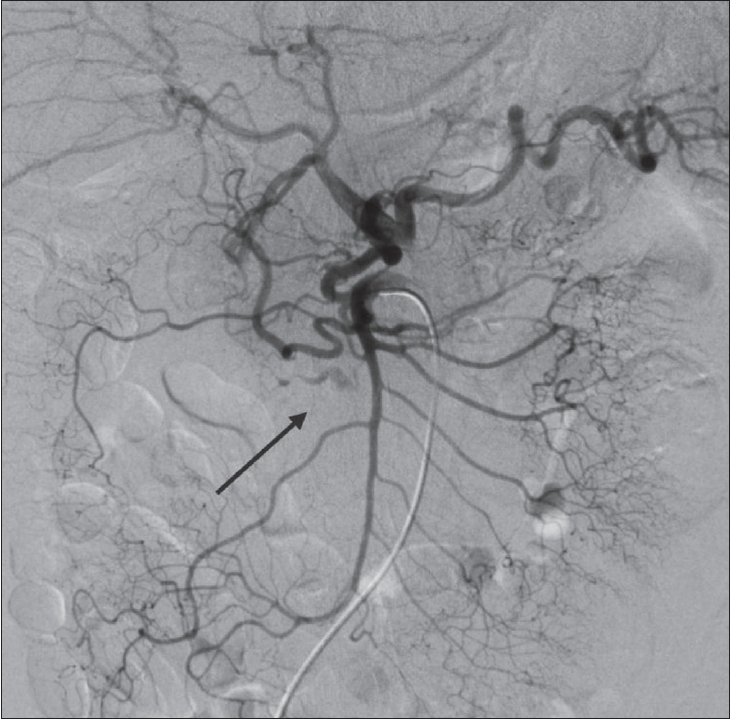
Superior mesenteric angiogram shows visualization of the hepatic and splenic arteries via collaterals, suggesting celiac axis stenosis. The ruptured aneurysm is observed as an arterial malformation in the anterior pancreaticoduodenal arcade (arrow)

**Figure 5:**
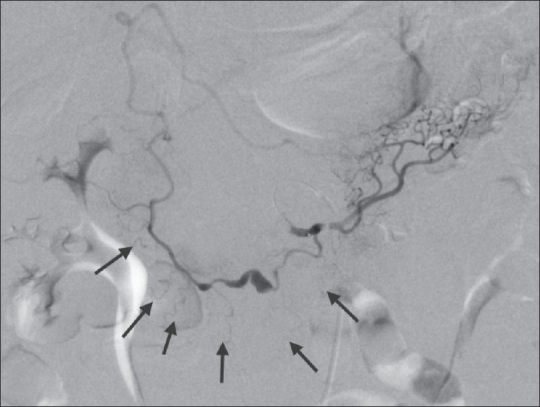
Selective angiogram of the anterior arcade via the superior mesenteric artery shows multi-segmental rupture of the aneurysm. Note that several lateral duodenal branches are communicating with the diseased segment of the artery (arrows)

A selective superior mesenteric artery angiogram showed retrograde flow in the pancreatic arcades and visualization of both the hepatic and splenic arteries [Figure 4]. A celiac artery angiogram revealed extravascular compression of the celiac axis, which was exacerbated during expiration, with partial relief during inspiration. This suggested a MAL as the most likely cause of the celiac axis stenosis. A selective angiogram of the pancreaticoduodenal arcade, obtained by placing a 2.4-Fr microcatheter (Microferret-18; Cook, Bloomington, IN) in the proximal pancreatic arcade, showed multisegmental arterial malformation with leakage of contrast media in the anterior arcade. Several duodenal branches were diverted from the diseased segment of the ruptured artery [Figure 5]. We decided that the best approach would be to occlude the entire aneurysm with the liquid embolic agent N-butyl cyanoacrylate (NBCA) using flow control techniques, reasoning that insufficient isolation of the ruptured aneurysm without termination of any lateral arterial communications to the ruptured aneurysm might increase the risk of recurrent bleeding.

**Figure 6:**
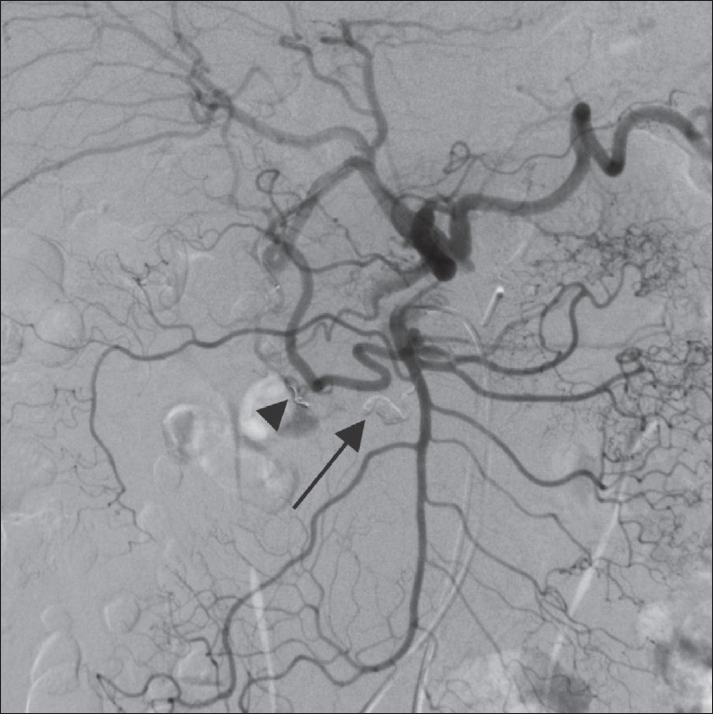
Superior mesenteric angiogram after injection with N-butyl cyanoacrylate (arrow) and distal coil placement (arrowhead) shows no visualization of the ruptured aneurysm

Using a contralateral femoral puncture, we inserted another 2.4-Fr microcatheter coaxially via the celiac axis into the distal part of the anterior pancreaticoduodenal arcade; through this, a 3-2 mm Tornado platinum detachable microcoil (Cook, Bloomington, IN) was placed in the distal aspect of the ruptured aneurysm to decrease the flow within the artery. Then, NBCA mixed with ethiodized oil (Lipiodol Ultrafluid; Andre Guerbet, Villepinte, France) in a 1:3 ratio was carefully injected through the catheter placed in the proximal aspect of the aneurysm via the superior mesenteric artery. The injection of the NBCA mixture was terminated when the whole aneurysm was visualized as a radiopaque glue cast. Angiography, following occlusion, confirmed complete embolization of the entire diseased artery, including the portion where the lateral branches diverted [Figure 6]. A follow-up CT scan on the seventh day showed hyperdense Lipiodol deposits in the hematoma. Another CT scan obtained 3 months following treatment showed a marked reduction in the size of the hematoma. The patient was taken up for surgical division of the MAL where it crossed anterior to the celiac axis. Although the surgical division of the MAL was successful, arteriography on the seventeenth day after surgery showed almost no regression of the celiac axis compression. We followed the patient with contrast-enhanced CT exams for possible recurrence of the aneurysm. During a follow-up period of 23 months, the patient has not presented with any further complaints of gastrointestinal discomfort, nor has he had any sign of retroperitoneal bleeding.

## Discussion

Pancreaticoduodenal artery aneurysms associated with the MAL syndrome develop secondary to the increased blood supply seen in the pancreaticoduodenal arcade to compensate for the reduced flow across the celiac axis.[1] Approximately 63-80% of patients with pancreaticoduodenal artery aneurysms exhibit celiac artery stenosis or occlusion.[4,5] The incidence of pancreaticoduodenal artery aneurysms has been estimated to be 3-18% in patients with the MAL syndrome.[6,7] Approximately 80% of pancreaticoduodenal arterial aneurysms are diagnosed after they rupture.[8] Mortality rates are reportedly higher in patients who have undergone surgery (19%) compared with patients treated by transcatheter arterial embolization (0%).[8,9]

During arterial embolization, the inflow and outflow vessels of the ruptured aneurysm are occluded. Selective catheterization with a coaxial system and embolization with coils provide definitive therapy in most reported cases.[3,4] Most failures are related to technical difficulties in catheterizing the target artery. In our case, narrowing of the proximal portion of the anterior pancreaticoduodenal arcade prevented optimum placement of the microcatheter. Instead, we were able to place microcoils distal to the aneurysm, via the celiac route. Residual communicating vessels may become alternative feeding arteries after isolation of the ruptured aneurysm. Ideally, complete packing of the whole diseased artery is preferable. NBCA can occlude the whole diseased segment along with other smaller feeders, and prior placement of the coil in the distal artery prevents the distal passage of the liquid embolic agent by decreasing the arterial flow. In our patient, no residual duodenal branches were observed after the entire impaired segment of the artery was packed. There was no sign of duodenal infarction, probably due to the development of a collateral circulation via the posterior branch of the pancreaticoduodenal artery.

In patients with pancreaticoduodenal artery aneurysms caused by celiac axis stenosis associated with the MAL syndrome, it is important to resolve the celiac stenosis to prevent recurrence. The treatment consists of surgical division of the MAL[10] or endovascular angioplasty.[11] Our patient underwent simple sectioning of his MAL since the results with endovascular angioplasty were not very satisfactory. Unfortunately, this surgical repair did not improve the celiac axis stenosis, partly because postoperative adhesions may have hampered decompression of the celiac axis.

In conclusion, isolation of a multisegmental rupture of a pancreaticoduodenal artery aneurysm possessing lateral arterial communication has the potential risk of re-bleeding. Alternatively, packing of the whole ruptured aneurysm by transcatheter embolization using NBCA, with distal placement of metallic coils, is an effective approach to decrease the risk of recurrent bleeding.
